# Data-driven modelling captures dynamics of the circadian clock of *Neurospora crassa*

**DOI:** 10.1371/journal.pcbi.1010331

**Published:** 2022-08-11

**Authors:** Amit Singh, Congxin Li, Axel C. R. Diernfellner, Thomas Höfer, Michael Brunner

**Affiliations:** 1 Heidelberg University Biochemistry Center, Heidelberg, Germany; 2 Theoretical Systems Biology [B086] Deutsches Krebsforschungszentrum, Heidelberg, Germany; Pázmány Péter Catholic University: Pazmany Peter Katolikus Egyetem, HUNGARY

## Abstract

Eukaryotic circadian clocks are based on self-sustaining, cell-autonomous oscillatory feedback loops that can synchronize with the environment via recurrent stimuli (zeitgebers) such as light. The components of biological clocks and their network interactions are becoming increasingly known, calling for a quantitative understanding of their role for clock function. However, the development of data-driven mathematical clock models has remained limited by the lack of sufficiently accurate data. Here we present a comprehensive model of the circadian clock of *Neurospora crassa* that describe free-running oscillations in constant darkness and entrainment in light-dark cycles. To parameterize the model, we measured high-resolution time courses of luciferase reporters of morning and evening specific clock genes in WT and a mutant strain. Fitting the model to such comprehensive data allowed estimating parameters governing circadian phase, period length and amplitude, and the response of genes to light cues. Our model suggests that functional maturation of the core clock protein Frequency causes a delay in negative feedback that is critical for generating circadian rhythms.

## Author summary

Circadian rhythms are endogenous autonomous clocks that emancipate daily rhythms in physiology and behavior. Lately, a large body of research has contributed to our understanding of clocks’ genetic and mechanistic basis across kingdoms of life, i.e., mammals, fungi, plants, and bacteria. Several mathematical models have made key contributions to our current understanding of the design principles of the *Neurospora crassa* circadian clock and conditions for self-sustained oscillations. However, previous models uncovered and described the principle properties of the clock in generic manner due to a lack of experimental data. In this study, we developed a mathematical model based on systems of differential equations to describe the core clock components and estimated model parameters from luciferase data that capture experimental observations. We demonstrate the model predictive control simulation emphasizing the importance of functional maturation of the core clock protein Frequency in generating circadian rhythms.

## Introduction

Circadian clocks orchestrate daily cycles of biochemical, physiological and behavioral processes. Anticipation and adaptation to recurring environmental changes is thought to improve the fitness of organisms [1,2,3]. Circadian clocks share specific characteristics that are crucial for their function: (1) Circadian clocks generate in the absence of external stimuli a self-sustaining rhythm of about 24h. (2) They respond to recurring external stimuli such as changes in temperature, light, and nutrient sources to synchronize with the 24-hour environmental day-night cycle. (3) Circadian clocks are temperature compensated, such that the period length of circadian rhythms is over a broad range not significantly affected by the average daily temperature [4,5,6]. Misalignment of the circadian clock and the environment contributes to several biochemical and physiological disorders including insomnia, mood disorder, diabetes, and cancer [7,8,9,10]. Circadian clocks of eukaryotes are based on cellular transcriptional-translational feedback loops (TTFLs) regulating the expression of core clock genes as well as clock-controlled genes [1,11,12]. In *Neurospora crassa*, the hetero-dimeric transcription activator White Collar Complex (WCC) and its inhibitor FFC, a complex containing Frequency (FRQ), FRQ-interacting RNA-helicase (FRH) [13,14] and casein kinase 1a (CK1a) [15] are the core components of the TTFL (see below). Previously, several mathematical models of the *Neurospora* circadian clock have been built on the basis of the core negative feedback loop constituted by the WCC and FRQ (FFC). Due to the unavailability of comprehensive experimental data these models uncovered and described principle properties of the clock in a rather generic manner, in most cases, not include detailed molecular interactions and mechanisms [16,17,18,19,20,21,22,23,24,25,26,27,28,29]. In this study, we analyzed a *WT* and a mutant strain Δ*vvd* [30], which is compromised in its capacity to adapt to light. We collected a comprehensive set of clock-related data by measuring *in vivo* in constant darkness and in light-dark cycles the expression of luciferase reporters of the core clock gene *frq* or *vvd*. In addition, we measured a luciferase reporter of *conidial separation-1 (csp-1)*. *csp1* is expressed in the subjective morning under the control of the WCC. CSP1 is a short-lived morning-specific repressor, and therefore, expression of its target genes peaks in the subjective evening [31]. Finally, we measured one of CSP-1’s target genes, *fatty acid metabolism-3 (fam-3)* [31].

The data warranted building a complex mathematical model with rather detailed molecular interactions. The data-driven model allowed us to estimate not only the expression phase but also the amplitude of rhythmically expressed genes and to uncover promoter-specific properties that determine their function in dark and light. Our approach demonstrates how high-resolution data sets can be used to more optimally exploit the theoretical capabilities of mathematical modeling.

## Results and discussion

### Interaction network of the *Neurospora* clock

**Fig 1:**
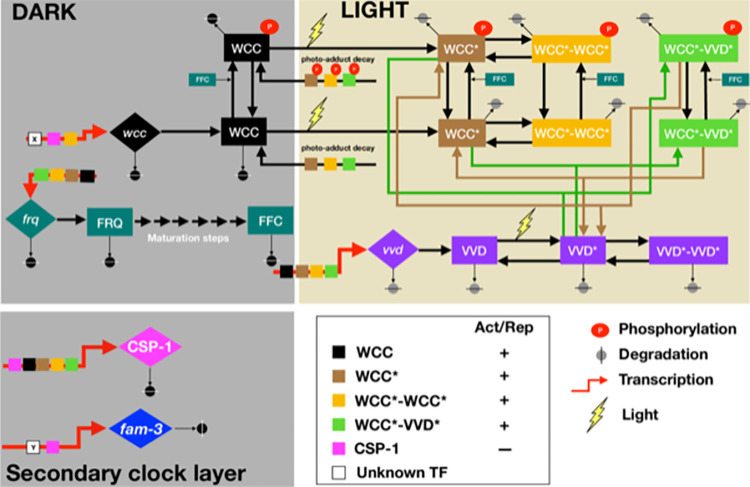
Schematic of the *Neurospora* circadian clock in dark and light.

To understand how the manifold molecular interactions implicated in the circadian clock of *Neurospora crassa* control autonomous circadian oscillations and entrainment, we established an interaction network based on the available data (Fig 1): White Collar-1 (WC-1) and White Collar-2 (WC-2) are PAS (PER- ARNT-SIM) domain containing GATA-type zinc finger proteins, which constitute the heterodimeric White Collar Complex (WCC), the core transcription activator of the circadian clock of *Neurospora* [32,33,34,35,36]. In the dark, transcription of *wc-1* and *wc-2* are controlled by unknown TFs [37]. We modeled transcription of wcc being equivalent to the transcriptional production of its limiting component *wc-1*. The subsequent translation and assembly of the WC-1 and WC-2 subunits constituting the WCC was described by a single production term.

Thick red arrows indicate transcription, and the small colored square boxes on these arrows indicate the TFs controlling the respective gene. White boxes X and Y indicate unidentified TFs activating transcription of *wcc* and *csp-1*, respectively. Large diamond and square boxes represent the indicated mRNA and protein species, respectively. FRQ, inactive Frequency protein; FFC, assembled, active FRQ-FRH-CK1a complex; WCC, White Collar Complex, VVD, Vivid; WCC and VVD, light-activated species; *csp-1*, *conidial separation 1; fam-3*, *fatty acid metabolism-3 (desaturase)*.

The WCC controls transcription of the core clock gene *frequency (frq)* [38]. FRQ protein was modeled as being initially inactive and unstable. FRQ homo-dimerizes [39], assembles with FRH [40], and CK1a [15], forming the active FFC complex [41]. The FFC and/or individual subunits or subcomplexes shuttle into the nucleus, receiving potentially licensing phosphorylation by CK1a [15,42] and/or by other kinases [43,44,45,46,47,48]. As little molecular detail is known on assembly and maturation, we modeled these processes in a generic manner by a linear chain of six consecutive steps. The FFC is the core negative element of the TTFL. It interacts transiently with and inactivates the WCC by facilitating its phosphorylation by the CK1a subunit of the FFC [49], and hence, we modeled the FFC acting enzymatically on the WCC and converting it into its phosphorylated, inactive form, P-WCC. As the WCC controls morning-specific transcription of *frq*, its inactivation closes the circadian negative feedback loop. Inactivated phosphorylated WCC is stable and accumulates at elevated levels [32,1,50,51,11,52,41]. In the course of a circadian period FRQ is progressively phosphorylated triggering its inactivation and degradation [15,53]. Inactivation of the FFC and degradation of its FRQ subunit was described by a single degradation/irreversible inactivation rate of the FFC. With decreasing amounts of active FFC, WCC is reactivated by dephosphorylation [49,13] and replenished by de novo synthesis, and then a new circadian cycle begins with reactivation of *frq* transcription.

Our wiring schematic of the core circadian oscillator in the dark (Fig 1, upper left box) is topologically equivalent to the Goldbeter limit cycle oscillator model [54]. However, the Goldbeter model as well as other generic circadian oscillator models [16,17,18,20,26] used a Hill-function for the transcriptional production of the core circadian inhibitors, PER in animals and FRQ in fungi, respectively, with Hill coefficients ranging from 2 to 7 [20,22,25,27]. While the Hill-coefficients in these models helped generate robust oscillations, molecular interactions underlying such highly cooperative processes are not known. To attain smaller Hill exponents several previous models introduced a time delay by including intermediate steps in the negative feedback loop [55,56,57]. A natural time delay is provided by the maturation of newly synthesized, inactive FRQ to active FFC, allowing us to describe WCC-activated transcription of *frq* by a simple Michaelis-Menten-like equation without introducing a Hill-coefficient. The WCC controls rhythmic expression of many clock-controlled genes, among them *vivid (vvd)* and *conidial separation-1 (csp-1)* [58,30,59,60], which were included in our model.

Light cues directly activate the WCC and thereby reset the circadian clock and induce several cellular processes including biosynthesis of carotenoids, asexual conidiospores formation, and development of sexual structures [61,62,63,31,64,60]. The light-activated WCC is a potent transcription activator of *frq* and *wc-1* (*wcc* in our model) as well as of many light-responsive genes including *vvd* and *csp-1* [65,58,30,60]. The WC-1 subunit of the WCC and VVD are blue-light photoreceptors harboring a flavin-binding light-oxygen-voltage-(LOV) domain (for review see [66,67]). VVD has no known function in the dark. Upon exposure to blue-light, a photo-adduct between a conserved cysteine residue of the LOV-domain and its bound FAD cofactor is formed [68]. WC-1 is activated in corresponding fashion [69,30]. The photo-adducts stabilize a conformation of the LOV domains that favors highly dynamic homo- and heterodimerization of VVD* and WCC* [30,68]. The light-activated WCC homodimer (WCC*WCC*) binds to light-response elements (LREs) [58]. WCC*WCC* was modeled as a potent transcription activator of *frq*, *vvd*, *wcc (wc-1)*, and *csp-1* [65,58,30,59,60]. As the interaction of VVD* with WCC* leads to photoadaptation of light-dependent transcription, the activity of the WCC*VVD* heterodimer and of monomeric light-activated WCC* was modeled as being equivalent to the activity of the dark form of the WCC. Light-activation triggers rapid hyperphosphorylation and accelerated degradation of WCC* [37,34,70,71] and thereby also affects levels of its readily equilibrating complexes, WCC*WCC* and WCC*VVD*. The light-activated, unphosphorylated WCC* is unstable and rapidly degraded [37,34,70,71], while interaction with VVD* stabilize the WCC*. The light-induced phosphorylation of WCC* was not explicitly modeled but is included into the higher degradation rate of light activated species, which was constraint in our model to a half-time ≤ 3 h.

In contrast, the FRQ dependent phosphorylation, inactivation and stabilization of all WCC species [49] was explicitly modeled. The LOV-domain photo-adducts of WCC* and VVD* decay spontaneously into their dark forms with a half-time in the range of hours [68,30]. All molecular reactions in the model were translated to a mathematical form describing either Michaelis-Menten or simple kinetics equations. The model contains 18 variables including mRNA and protein species. The detailed mathematical equations are displayed in S1 Text. The model includes the key features of the circadian clock and is sufficiently complex to allow an adequate, and mostly quantitative, molecular interpretation of experimental data.

### High amplitude response to light versus low amplitude dark oscillations

**Fig 2:**
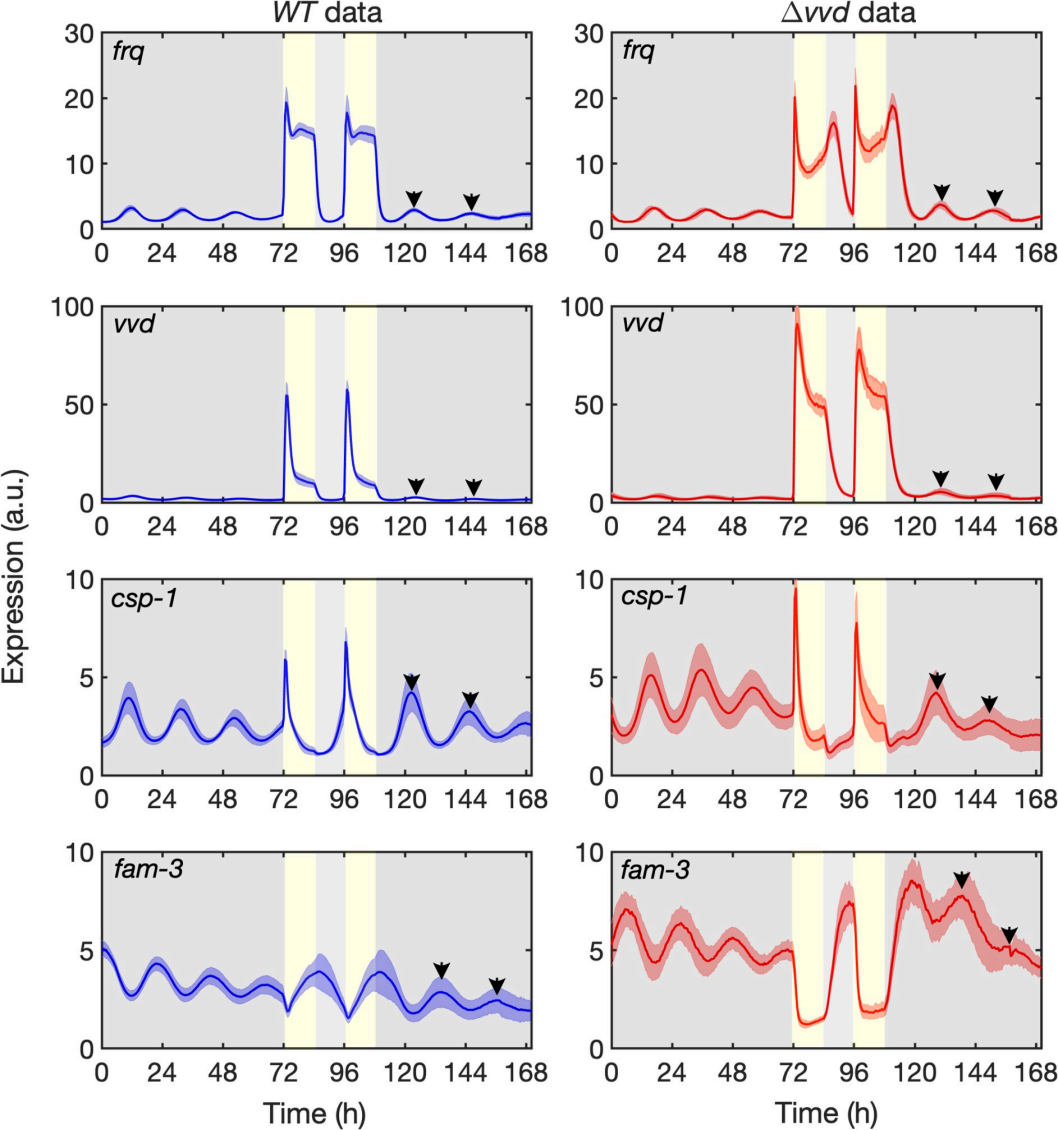
Temporal expression profiles of reporter genes in DD and LD. Luciferase activity under the control of the indicated promoters in *WT* (left panels) and Δ*vvd* (right panels). The solid line represents the average of 30 measurements from three independent experiments. The shaded areas correspond to the standard deviation, SD. Yellow vertical boxes and grey areas indicate 12h light periods and dark periods, respectively.

In order to collect sufficient data as a basis for the molecular model of the circadian clock we generated *WT* and Δ*vvd* reporter strains expressing destabilized luciferase (lucPEST) [72] under the control of the morning-specific *frq*, *vvd* and *csp-1* promoters and the evening-specific *fam-3* promoter. The *lucPEST* transcription units carried the 3’ region of the *trpC* gene of *Aspergillus nidulans* for termination of transcription and the reporter genes were inserted downstream of the *his-3* locus into the genome of *Neuropora*. Mycelial cultures of these strains were grown in 96-well plates (with more than 30 replicates in 3 independent experiments) on solid agar medium. The medium contained sorbose in order to restrict growth, which allows live recordings of bioluminescence over many days. The cultures were grown on the sorbose medium and synchronized by exposure to 12h light, 12h dark and 12h light and then transferred to dark for 24h before the luciferase measurement was started (t = 0) (Fig 2). After 3 days in constant darkness (t = 72h) the samples were exposed to 12h light, 12h dark and 12h light, and then kept in the dark for another 64h. In constant darkness the expression levels of all *lucPEST* reporters oscillated but showed dampening over time (see Fig 2). It is not clear whether and to what extent the dampening is the consequence of desynchronization of individual nonconnected hyphae or due to a real reduction of amplitude of the oscillator. The expression of *frq*, *vvd*, and *csp-1* reporters oscillated with peaks levels in the subjective morning, while *fam-3*, which is repressed by CSP-1 in the morning, was rhythmically expressed in antiphase with peak levels in the subjective evening. After light was turned on, expression of *frq* was rapidly induced in *WT* to a level ~18-fold higher, but then dropped and adapted to a level ~12-fold higher than in the dark. Expression of *vvd* was rapidly induced ~50-fold compared with the level in the dark and then adapted to a ~10-fold higher level, and *csp-1* was induced ~6 and fully adapted to its dark expression level. Thus, responses of *frq*, *vvd*, and *csp-1* to light had much larger amplitudes than autonomous oscillations in constant darkness. Moreover, the three light-inducible promoters responded with different inactivation kinetics to light-activated WCC. In contrast, the evening-specific *fam-3* reporter was transiently repressed after the light was turned on. This is consistent with the light-induced transient expression of CSP-1, which represses its own gene, *csp-1*, as well as *fam-3* and many other genes [31].

As expected on the basis of previous studies [72,73,74,75], all oscillations were phase-delayed in Δ*vvd* (Fig 2, right panels, S1 Fig). The transcriptional dynamics in light-dark cycles of *frq* and *vvd* were quite different in Δ*vvd* as compared to *WT*. The light response of the *vvd* reporter was qualitatively similar to that in *WT*. However, the spike level after light on was almost twice that of *WT*, and the light-adapted expression level was ~5-fold higher than in *WT*. These data are consistent with the increased activity of light-activated WCC in the absence of its light-dependent inhibitor, VVD [30]. In contrast, the initial light-induced spikes of the *frq* reporter were similar in Δ*vvd* and *WT*, suggesting that the *frq* promoter was already functionally saturated at *WT* levels of light-activated WCC. After the initial light-induced spike, *frq-lucPEST* expression levels decreased sharply and more markedly in Δ*vvd* than in *WT*, and then increased again in the further course of the light phase. When light was turned off, *vvd* transcription decreased as expected, consistent with the decreasing level of light-activated WCC. Surprisingly, however, the *frq* level in Δ*vvd* increased transiently after the lights were turned off and then dropped. The difference in expression dynamics of *frq* compared with *vvd* after light on and even more so after the light-to-dark transition is consistent with the previously reported refractory behavior of the frq promoter [76,77]. Indeed, in Δ*vvd* the *frq* promoter is partially repressed in light, and hence not maximally active. The rapid decrease of the light-induced transcription spike after light on reflects the dynamics of the light-dependent partial repression. Similarly, the transient increase in *frq-lucPEST* transcription in Δ*vvd* after turning off the light is consistent with the repression of the *frq* promoter being relieved faster than the WCC activity (level) decreases.

### The model quantitatively captures circadian oscillations and light entrainment in *WT Neurospora*

Our model was trained to the temporal expression profiles of the *frq*, *vvd*, *csp-1* and *fam-3* reporters in *WT* and Δ*vvd*. The parameter space (66 kinetic parameters and 64 initial condition) was restricted to a biological meaningful range as described in S1 S1 Text and S2 Table. The parameters were estimated using maximum likelihood (lsqnonlin optzimizer) implemented in D2D/Matlab [78].

**Fig 3:**
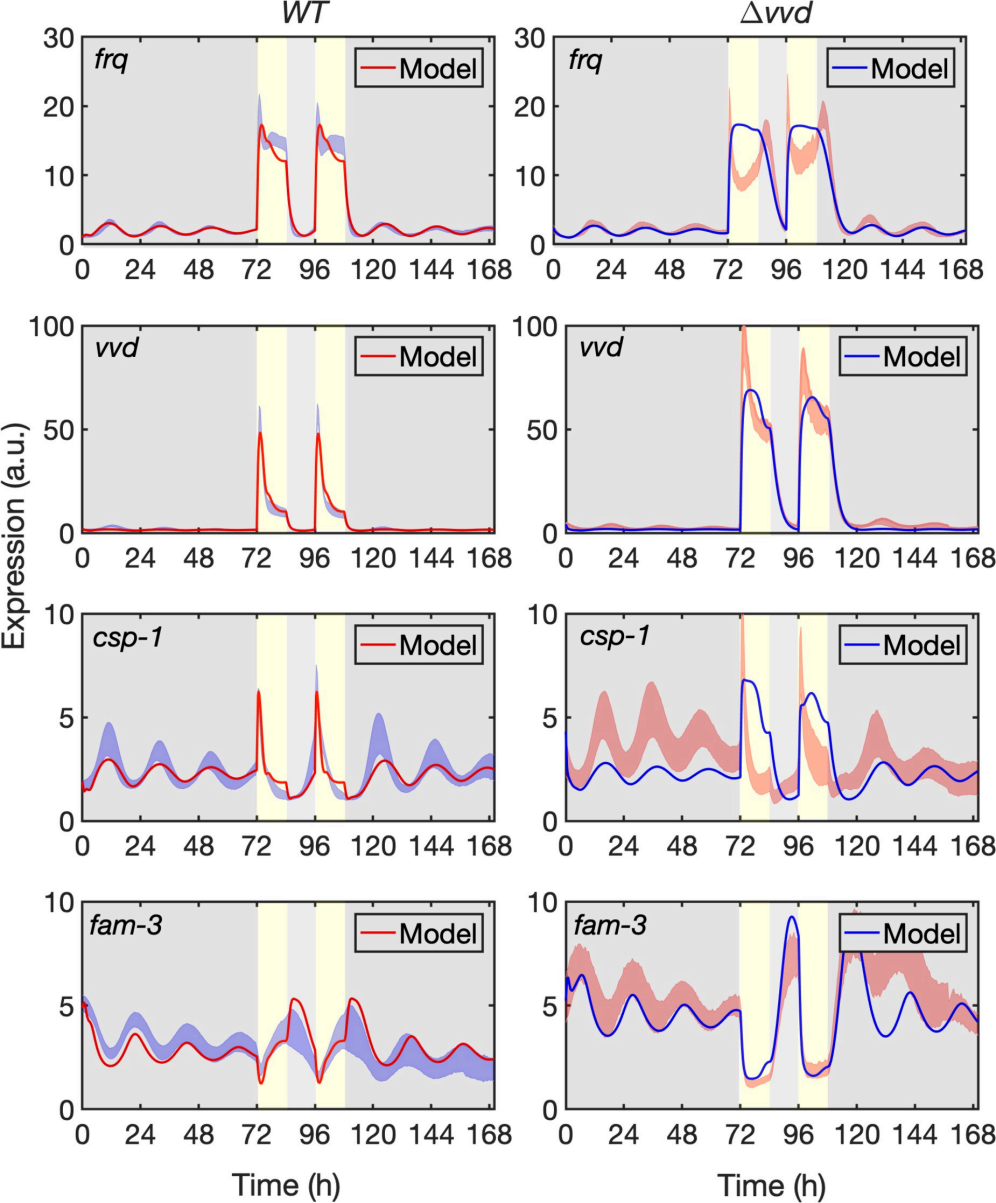
Model fitted to reporter gene expression in DD and LD.

Overall, our model simulations show oscillation of all reporters in the dark and responded in appropriate manner to the LD-cycles (Fig 3). The simulations of *frq* and *vvd* transcription in *WT* captured the experimental data quite well, reproducing period length, phase and amplitude of the free-running oscillations as well as the transcription dynamics in LD cycles (Fig 3, left panels). The simulations of *csp-1* expression in WT captured period length and phase in DD and part of the dynamics in LD cycles, while amplitude of the model deviated somewhat from the measured data. In our model, we assumed that *csp-1* is rhythmically activated by the WCC and rhythmically repressed by CSP-1. The deviation of data and model could indicate that, in addition to WCC, an unknown transcription activator contributes to the *csp-1* expression. Furthermore, compared to the luciferase data, the model predicted slightly higher *csp-1* levels towards the end of the 12h light phases, and the level then dropped abruptly after lights-off in the model. This sudden drop in *csp-1* RNA, and hence of the short-lived CSP-1 repressor [31], lead to a rapid relieve of *fam-3* repression and thus, the model showed transiently slightly higher *fam-3* levels than experimentally observed.

The simulations of gene expression in Δ*vvd* (Fig 3, right panels) faithfully reproduced the period length and phase of all reporters and captured their phase delays compared with *WT* (S1 Fig). The amplitudes of *frq* and *vvd* oscillations in the dark were modeled quite well. In contrast the amplitude of the *csp-1* oscillation was underestimated even more than in *WT*, supporting the notion (see above) that the *csp-1* transcription could be additionally supported by an unknown activator. The expression dynamics of vvd in LD cycles were fully captured, suggesting that Michaelis-Menten kinetics is suitable to quantitatively describe the activity of the *vvd* promoter in *WT* and in Δ*vvd*. The higher light-induced expression level of the *vvd* reporter in Δ*vvd* compared with *WT* was also captured by our model. Because the transcriptional output of the *vvd* promoter is very sensitive to the activity of the light-activated WCC [76], the agreement of our model with the Δ*vvd* and *WT* data justifies our differentiation of various light-activated WCC species (WCC*, WCC*-WCC* and WCC*VVD*) and the rates of their interconversion. Our model did, however, not capture the complexity of the expression profile of *frq* in light-dark cycles, indicating that Michaelis-Menten-like promoter activation was not sufficient to describe the response of the *frq* promoter to light cues, in particular in Δ*vvd*. Specifically, in Δ*vvd*, our Michaelis-Menten-based model of the *frq* promoter produced almost square-waves of *frq* expression in LD with a rather slow decline after light was turned off. In contrast, the luciferase measurements revealed an initial overshoot of *frq* expression after light on, followed by rapid adaptation of *frq* in continuous light and then a transient increase in *frq* expression after the LD transition. As discussed above, the complex transcription dynamics reflect the previously reported partial repression of the light-activated *frq* promoter by high levels of activated WCC [76,77]. This particular feature of the *frq* promoter has not yet been included in our molecular model scheme as the underlying mechanism is still awaiting identification and functional characterization of the components involved. However, it is only the acquisition of high-resolution data that made it possible to reveal the discrepancy between model and data and thus predict that the transcriptional dynamics of the *frq* promoter in response to light cannot be modeled by simple Michaelis-Menten-like promoter activation. Such a prediction is not possible with generic models.

Expression of *csp-1* in the Δ*vvd* background increased sharply after light on and then adapted rapidly, reflecting the negative feedback of CSP-1 on its own transcription. The dynamics of the CSP-1 feedback, which was captured by the model in *WT*, was underestimated in Δ*vvd*, indicating that the model did not quantitatively describe the enhanced light-induced expression of CSP-1 in absence of VVD. Potentially, Michaelis-Menten-like promoter regulation is not sufficient to describe the dynamics of the *csp-1* promoter.

Trajectories of the model (solid red lines in *WT* and solid blue lines in *Δvvd*) fitted to the luciferase activity expressed under the control of the indicated promoters (standard deviation is shown, see Fig 2) in *WT* (left panels) and *Δvvd* (right panels). Yellow vertical boxes and grey areas indicate 12h light periods and dark periods, respectively.

We then challenged the model by simulating 10 consecutive light-dark cycles (S2 Fig, left panels). The system responded in the same manner to each of the LD cycles. None of the components was depleted or accumulated to higher levels over the time period, indicating that the system was balanced. In constant darkness the expression levels of all *lucPEST* reporters oscillated but showed dampening over time (see Fig 2). Our model was trained on the dampening bioluminescence oscillations in the dark it reproduced the dampening (Fig 3). Indeed, simulating prolonged incubation in the dark led to a substantial loss of amplitude (S2 Fig, right panels). Since we have not allowed the possibility of desynchronization of individual oscillators in our analytical model, the damping in our model is due exclusively to a gradual loss of amplitude. However, it is possible that the experimentally observed dampening is due to desynchronization and that the modelled loss of amplitude does not reflect a physiological relevant process. Indeed, loss of amplitude was prevented when the dephosphorylation rate of WCC, i.e. its reactivation, was slightly increased (S3 Fig). Overall, however, the model almost quantitatively predicted the transcriptional dynamics of two hierarchical levels of the circadian clock in the dark and in light-dark cycles in *WT*. The model was generally less precise in predicting transcription rhythms and dynamics in Δ*vvd*.

To validate the predictive power of the model, we trained the model without using the *csp-1* and *fam-3* data from the vvd strain. The model reproduced the training data and predicted the *csp-1* and *fam-3* dynamics in the Δ*vvd* strain (S4 Fig). It should be noted that due to the high-dimensional parameter space and oscillatory nature of the model, a single best-fit parameter set was difficult to pinpoint (S5 Fig). In fact, multiple best-fit parameter sets can exist even for low-dimensional models [79]. However, further analysis of the top 10 best-fit parameter sets showed that the parameter values were largely constrained by the experimental data, rather than freely variable (S5 Fig), indicating that our model is well informed by the data.

The detailed data-based model allowed us to ask specific questions that cannot be addressed with a generic clock model. For example, previous models of the *Neurospora* circadian clock used Hill functions which was crucial for robust circadian oscillation of *frq* transcription [16,17,18,24,25,27].

**Fig 4:**
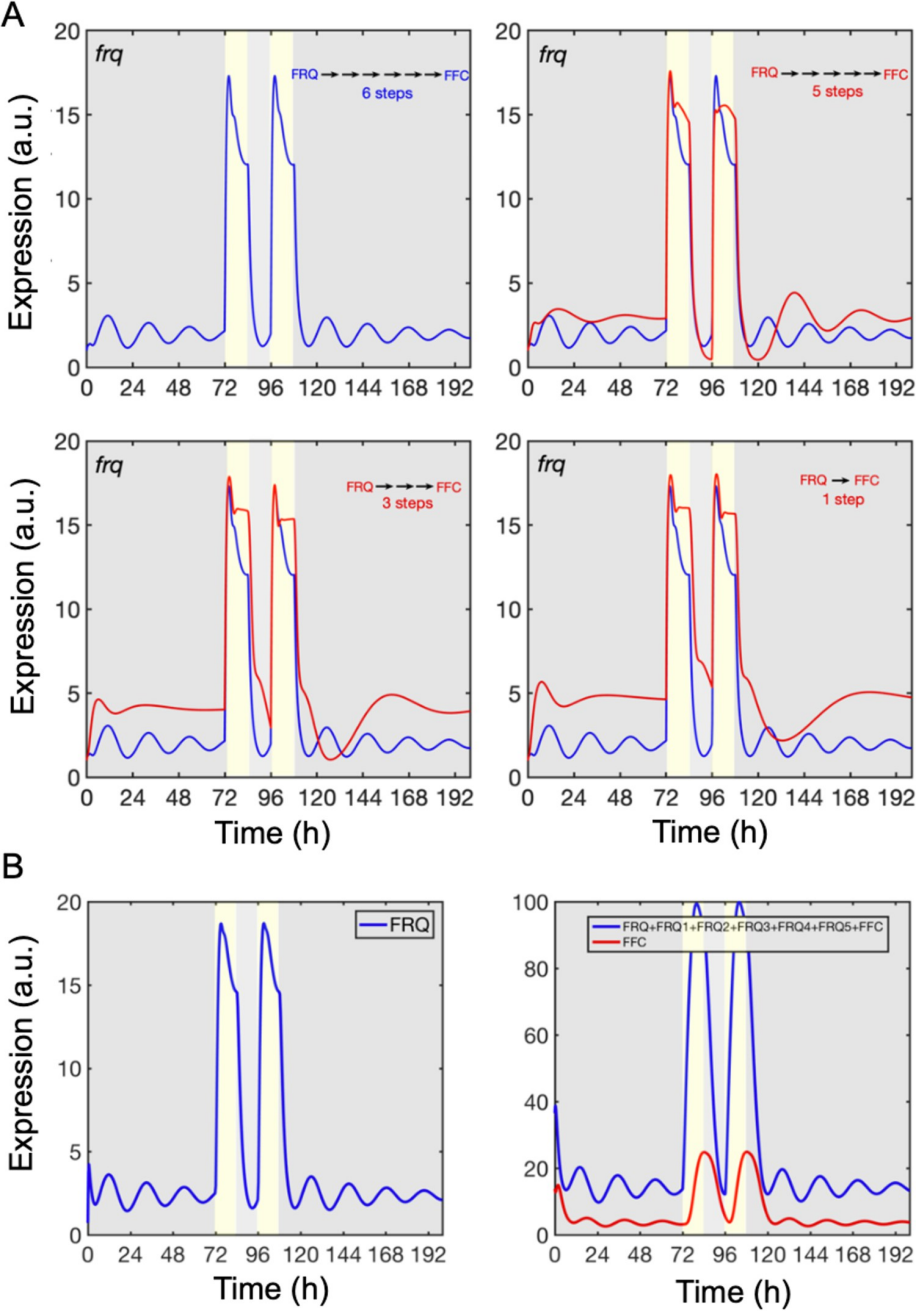
Maturation of inactive FRQ to active FFC is crucial for circadian rhythmicity. (A) Maturation of FRQ to FFC was modelled by a linear chain of 6 generic steps. The impact of the number of steps on *frq* expression levels and rhythm is shown. (B) Delay between newly synthesized FRQ and assembled FRH (left panel) and abundance of all unassembled FRQ-species versus assembled FRH (right panel).

As we did not introduce a Hill-function for the transcriptional production of *frq*, the circadian oscillation in our model depends critically on the delay between the synthesis of *frq* and the appearance of the fully assembled and active FFC. This process, which was modeled by FRQ translation and six generic maturation steps, introduced a delay in the accumulation of active FFC and supported circadian oscillation of *frq*. Shortening the delay by successive removal of maturation steps resulted in increasing expression levels of *frq* and arrhythmicity (Fig 4A). Thus, our model suggests that maturation of the FFC may be an important process that should be studied experimentally. Indeed, this aspect is still very poorly understood, and we do not know how, when, in what order, and in what cellular compartments FRQ dimerizes, assembles with FRH and CK1a, and if FRQ requires licensing phosphorylation somewhere along this pathway to become an active inhibitor of WCC in the nucleus. CK1a is anchored to FRQ and slowly phosphorylates FRQ at many sites, which may be part of a delayed activation mechanism of FFC [80,15,81,82]. Our model also predicts that only a fraction of the newly synthesized FRQ assembles with FRH while the majority is degraded. Due to the slow assembly process unassembled and partially assembled FRQ is more abundant than fully assembled, active FFC at any given time (Fig 4B). The high fraction of partially assembled FRQ species could explain the substoichiometric levels of FRH that are found in complex with total FRQ in vivo [83], although one molecule of FRQ is capable of binding one molecule of FRH [84].

**Fig 5:**
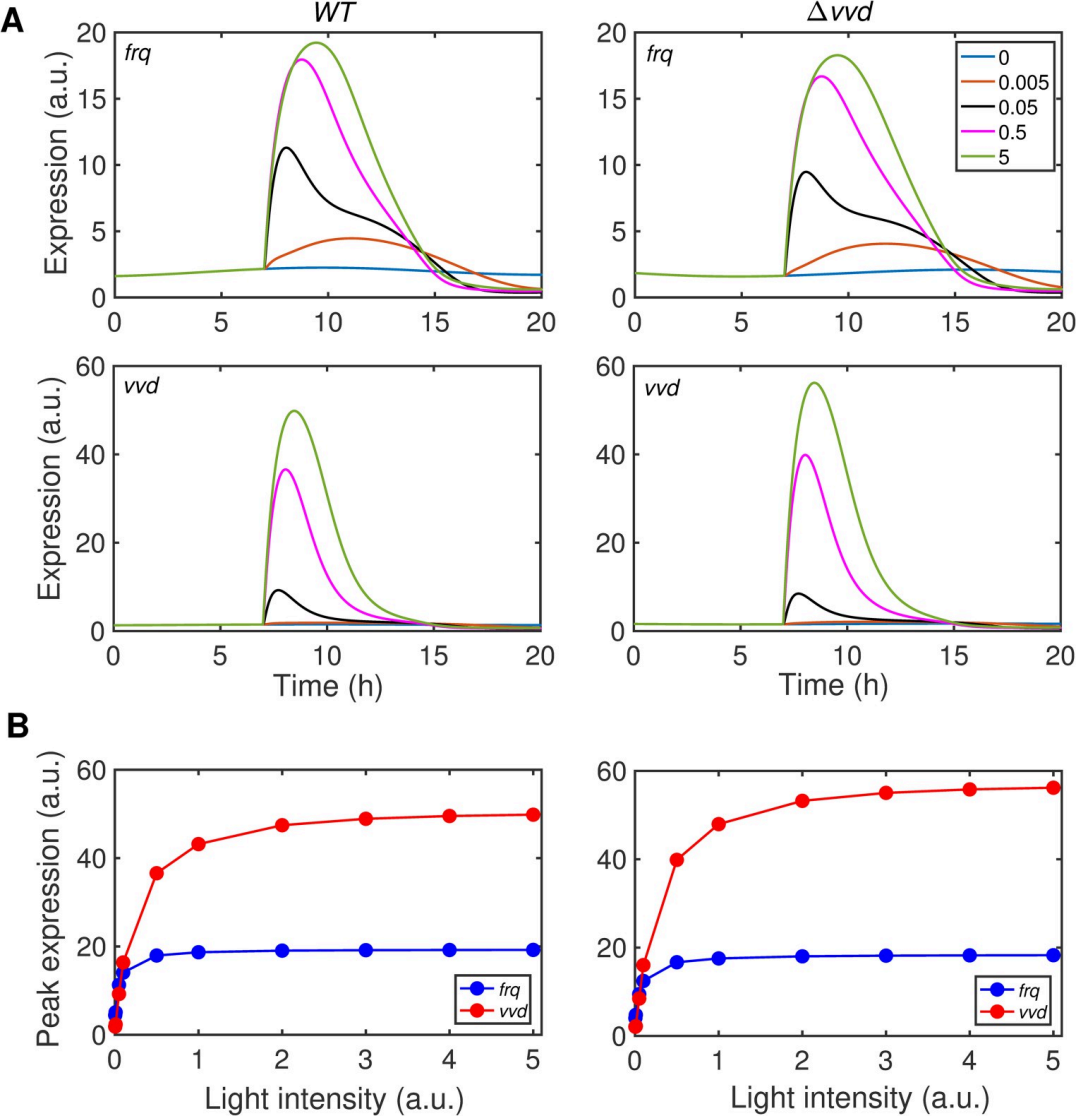
Modelling the response of *frq* and *vvd* promoters to light-pulses of different intensity. *WT* (left panels) and *Δvvd* (right panels) were exposed at t = 7 min to a virtual LP. (A) Modeled response of *frq* and *vvd* to virtual LPs of the indicated intensities. (B) Plot of peak expression levels of *frq* and *vvd* versus LP intensity. Simulated LP intensities: 0; 0.005; 0.005; 0.05; 0.5 and 5.0 arbitrary units (a.u.).

The continuous recording of promoter-specific luciferase reporters *in vivo* in dark and light made it possible to estimate the intrinsic maximum transcription rate of the *frq* and *vvd* promoters (v_max_) and to uncover functional differences in promoter architecture. Previous quantitative ChIP-PCR analyses revealed a similar affinity of the light-activated WCC for the LREs of *frq* and *vvd* [76,77]. Yet, our model predicted a small K_M_ for WCC-dependent transcription activation of *frq* and a larger K_M_ for *vvd*. The data indicate that the affinity of the transcription factor for its LRE does not directly correlate with gene transcription. The difference likely reflects that the *frq* LRE is located in the core promoter such that bound WCC*WWC* can directly activate the core promoter. In contrast, the *vvd* LRE is located in an upstream enhancer region. Hence, activation of *vvd* transcription is dependent on looping of the LRE-bound WCC*WWC* to the core promoter. The coupled equilibria of TF-binding to the remote LRE and looping of the LRE-bound TF to the promoter result in an overall higher K_M_ for the activation of transcription at the *vvd* promoter [76]. Furthermore, our model predicted a small v_max_ for *frq*, consistent with *frq* being a weak promoter with an intrinsically low maximal transcription rate. The light-activated *vvd* promoter has a high intrinsic maximal transcription rate, consistent with a large predicted v_max_. Our simulations with these promoter-specific parameters (Fig 5A and 5B) captured in principle the previously reported saturation of *frq* transcription at rather low light intensity while *vvd* responds over a much wider range proportional to the intensity of light [76,77].

In summary, the *luc* reporter strains allowed measuring transcript levels and dynamics with high temporal resolution. Obtaining such comprehensive data justified the construction of a detailed molecular model of the *Neurospora* circadian system. The wiring scheme of the model and the derived kinetic parameters captured crucial features of the circadian system, but cannot yet accurately predict the temporal dynamics of all processes. This discrepancy led to new testable hypotheses. Overall, therefore, to circadian clocks as the one presented here can become a valuable tool for quantitatively understanding the interaction of the molecular clock components. Modeling of circadian clocks based on measured data has been applied to serval other organisms, such as mammals [85,86,87], cyanobacteria [88,89]. We believe that data-based approaches are specifically suitable for facilitating a system-level understanding of circadian rhythms by integrating data from complex conditions such as light, temperature, nutrition, and distinct genetic backgrounds.

## Materials and methods

### *Neurospora* strains and plasmids

The *Neurospora* strains denoted with *WT* and Δ*vvd* carried the *ras1*^*bd*^ mutation (Belden et al., 2007) and either a p*frq-lucPEST*, p*vvd-lucPEST* (Cesbron *et al*., 2013), p*fam3-lucPEST*, formerly called desat-lucPEST (Sancar *et al*., 2011) or a p*csp1-lucPEST* reporter gene integrated downstream of the *his-3* locus. For p*csp1-lucPEST*, a 7395bp fragment immediately upstream of the csp-1 ORF was amplified from gDNA and cloned in front of the lucPEST ORF using EcoRI (vector)/MfeI(PCR insert) and NotI restriction sites. The resulting plasmid was used to transform the above mentioned *Neurospora* strains.

### Real-time luciferase activity measurements

Sorbose medium containing 1x FGS (0.05%fructose, 0.05% glucose, 2% sorbose), 1x Vogels, 1% agarose, 10 ng/ml biotin, and 25 μM firefly Luciferin was used for the assessment of the luciferase activity. 96-well plates were inoculated with 3 X 10^4^ conidia per well and incubated in DD at 25°C. Bioluminescence was recorded in DD or LD at 25°C with EnSpire Multilabel Readers (Perkin Elmer). The light intensity was 0.25 *μ*E. Three independent experiments with multiple biological replicates each were performed to generate the data (n≥30).

### Mathematical modelling and parameter estimation

The reactions of the model were translated to ODEs

$$\frac{d(X(t,\theta ))}{dt}=f_{x}(X(t,\theta ),u(t),\theta )$$

where ***θ*** is a parameter ***θ*** = (***θ*_1_**, ***θ*_2_**..***θ_i_***). The initial state of the system is described by $X(0,\theta )=f_{X_{0}}(\theta )$ The variables ***X*** correspond to the dynamics of the concentration of molecular components of the model. To derive the unknown model parameters, the circadian model was calibrated by a maximum likelihood estimation using quantitative experimental data obtained by luciferase measurements. The model was fitted to the luciferase data using (MATLAB version 2016b) D2D software package from http://www.data2dynamics.org [78].

## Supporting information

S1 Text Mathematical model. Equations of the underlying mathematical model and parameter estimation.
(PDF)

S1 Fig Phase delay of expression rhythms in *Δvvd*. Modelled trajectories of the expression rhythms in *WT* and *Δvvd* (see Fig 3) were superimposed.
(EPS)

S2 Fig Characterization of the model. (A)Simulated response of reporters to 10 repetitive LD cycles. (B) Rhythms of reporter genes dampen in constant darkness. Expression rhythms in the dark of the reporters were modelled for 400 h. Inserts show zoom-in of the expression rhythms between 350 and 400 h.
(EPS)

S3 Fig Impact of WCC dephosphorylation rate on *frq* expression rhythm. The modeled *frq* expression rhythm damps in the dark (blue trajectory). When the predicted dephosphorylation (reactivation) rate of WCC (k_d2_ = 0.18) was stepwise lowered dampening was reduced (k_d2_ = 0.14, red trajectory) or abolished (k_d2_ = 0.08, red trajectory) and the amplitude increased.
(EPS)

S4 Fig Model validation by predicting *csp-1* and *fam-3* expression in *Δvvd*. The model was trained without using the *csp-1* and *fam-3* expression data from *Δvvd*. The shaded areas correspond to the standard deviation of the expression data for the indicated reporters in *WT* (left panels) and *Δvvd* (right panels). The solid red and blue trajectories represent the best fit to the *WT* data (left panels) and *Δvvd* data (upper right panels) that were used for model training. The solid green trajectories (lower right panels) represent the prediction of the model for *csp-1* and *fam-3* expression dynamics in *Δvvd*. Yellow vertical boxes and grey areas indicate 12h light and dark periods, respectively.
(EPS)

S5 Fig Constraints of model parameters by the data. The top 10 best-fit parameter sets were selected based on the *χ*^2^ value (10 lowest *χ*^2^ values) from the final fits using the Latin Hyper Cube sampling method (700 random initial parameter sets). (A) Gene activities simulated by the top 10 best-fit parameter sets. Color code represents the ranking of the parameter sets. Yellow vertical boxes and grey areas indicate 12h light and dark periods, respectively. (B) Histograms and corresponding smoothed density plots for individual selected kinetic parameters. X-axes represent the parameter value in log10 scale. Y-axes represent the density of the histogram.
(TIFF)

S1 Table Model variables. (XLSX)

S2 Table Parameters and initial conditions. (XLSX)

S3 Table Luciferase reporter expression in *WT*. (XLSX)

S4 Table Luciferase reporter expression in *Δvvd*. (XLSX)
