# Supplementary material for: Data-driven modelling captures dynamics of the circadian clock of Neurospora crassa
Source: PLoS Comput Biol. 2022 Aug 11;18(8):e1010331. doi: 10.1371/journal.pcbi.1010331 (PMC9397904; doi:10.1371/journal.pcbi.1010331)
Supplement: S1 Text — Equations of the underlying mathematical model and parameter estimation. (PDF) [file pcbi.1010331.s001.pdf]

1 MATHEMATICAL MODEL

The detailed descriptions of the model variables and parameters are listed in S1 and S2 Table. The molecular interactions in the circadian clock of *Neurospora crassa* were translated into a set of ordinary differential equations. We used Michaelis-Menten type functions for mRNA production of *wcc, frq, vvd, csp-1, fam-3* and FRQ-mediated WCC phosphorylation; the rest reactions were described by mass action kinetics. We fixed some parameters during computational inference (highlighted in S2 Table), e.g. the degradation rate of FRQ, VVD, CSP-1, FAM-3, based on previously known experiment [1, 2, 3, 4, 5, 6]. The parameter search ranges are also provided in S2 Table. Further, fast reaction kinetic such as the conversion of monomer to dimer (WCC and VVD) are constrained according to previously published model [7].

1.1 WCC dynamics

The white collar-1 (WC-1) and white collar-2 (WC-2) proteins form White Collar Complex (WCC) via PAS domain[8]. WCCs sense blue light through the LOV domain of WC-1 and form homodimers, which bind to and activate many target genes [9, 10, 11, 12, 13]. Due to the presence of flavin adenine dinucleotide (FAD)-binding photoreceptor in WC-1, the activation of WCC differs in the dark to light conditions [11, 14, 15]. The VVD protein, which contains the LOV domain, behaves as a positive regulator and inhibitor on WCC and forms the WCC-VVD heterodimer complex in light [7]. Here, we modeled *wc-1* mRNA (assuming WC-2 is sufficiently abundant) and four distinct active forms of WCC protein, i.e., WCC expression in constant dark W_d, monomer form W_l, dimer form W_lW_l, and heterodimer complex form with VVD W_lV_l in light.

1.2 Dynamics of dark form WCC

We converted the dark-form WCC dynamics into three ordinary differential equations.

1. Basal level of mWCC activity in the dark, W_lW_l mediated transcription followed by CSP-1 inhibition
2. Translation of W_d protein and its degradation
3. Inhibition of W_d by FFC-mediated phosphorylation generating the negative feedback loop as well as dephosphorylation of W_d
4. Degradation of W_d and W_{dp}

$$\frac{d[mWCC]}{dt} = (K_{\text{basal}} + k_1 \frac{[W_lW_l]}{(K_1 + [W_lW_l])}) \frac{K_{\text{cp}_1}}{(K_{\text{cp}_1} + [CSP])} - k_{\text{d1}}[mWCC] \tag{1}$$

$$\begin{aligned}\frac{d[W_d]}{dt} &= k_2[mWCC] - k_{\text{pos1}} \frac{[W_d][FFC]}{(K_{\text{pos1}} + [W_d])} + k_{\text{dpos1}}[W_{dp}] - k_{\text{d2}}[W_d] \\ &+ k_4[W_lW_l] + k_5[W_lV_l] - l_1[light][W_d] + k_3[W_l]\end{aligned} \tag{2}$$

$$\begin{aligned}\frac{d[W_{dp}]}{dt} &= k_{\text{pos1}} \frac{[W_d][FFC]}{(K_{\text{pos1}} + [W_d])} - k_{\text{dpos1}}[W_{dp}] - k_{\text{dp1}}[W_{dp}] \\ &- l_1[light][W_{dp}] + k_3[W_{lp}] + k_5[W_{lp}V_l] + k_4[W_lW_{lp}]\end{aligned} \tag{3}$$

1.3 Dynamics of light form WCC

In the presence of light, dark form WCC transitions to the light-activated WCC, followed by FCC mediated phosphorylation.

1. Activation of dark form WCC by light (converting to light form WCC).
2. Inhibition of W_l by FFC-mediated phosphorylation generating the negative feedback loop and dephosphorylation of W_{lp}
3. Degradation of W_l and W_{lp}
4. Photo adduct decay of W_l and W_{lp}

$$\frac{d[W_l]}{dt} = l_1[light][W_d] - k_3[W_l] + k_7[W_lW_l] - k_6[W_l]^2 - k_{\mathrm{pos2}}\frac{[W_l][FFC]}{(K_{\mathrm{pos2}} + [W_l])} + k_{\mathrm{dpos2}}[W_{lp}] - k_{\mathrm{d3}}[W_l] + k_8[W_lV_l] - k_9[W_l][V_l] \tag{5}$$

$$\frac{d[W_{lp}]}{dt} = k_{\mathrm{pos2}}\frac{[W_l][FFC]}{(K_{\mathrm{pos2}} + [W_l])} - k_{\mathrm{dpos2}}[W_{lp}] - k_{\mathrm{dp2}}[W_{lp}] + l_1[light][W_{dp}] - k_3[W_{lp}] + k_8[W_{lp}V_l] - k_9[W_{lp}][V_l] - k_6[W_{lp}]^2 + k_7[W_lW_{lp}] \tag{6}$$

1.4 WCC homodimer dynamics

The light-activated WCC monomers dimerize and undergo phosphorylation regulated by FCC, which were modeled as follows.

1. Reversible dimerization of WCC (W_lW_l) in the presence of light
2. Inhibition of W_lW_l by FFC-mediated phosphorylation, generating the negative feedback loop and dephosphorylation of W_lW_{lp}
3. Photo adduct decay of W_lW_l and W_lW_{lp}
4. Degradation of W_lW_l and W_lW_{lp}

$$\frac{d[W_lW_l]}{dt} = k_6[W_l]^2 - k_7[W_lW_l] - k_4[W_lW_l] - k_{\mathrm{pos3}}\frac{[W_lW_l][FFC]}{(K_{\mathrm{pos3}} + [W_lW_l])} + k_{\mathrm{dpos3}}[W_lW_{lp}] - k_{\mathrm{d4}}[W_lW_l] \tag{7}$$

$$\frac{d[W_lW_{lp}]}{dt} = k_{\mathrm{pos3}}\frac{[W_lW_l][FFC]}{(K_{\mathrm{pos3}} + [W_lW_l])} - k_{\mathrm{dpos3}}[W_lW_{lp}] - k_{\mathrm{dp3}}[W_lW_{lp}] + k_6[W_{lp}]^2 - k_7[W_lW_{lp}] - k_4[W_lW_{lp}] \tag{8}$$

1.5 WCC-VVD heterodimer dynamics

1. WCC and VVD heterodimerize (W_lV_l) in the presence of light, forming a negative feedback loop.
2. Inhibition of W_lV_l by FFC-mediated phosphorylation generating the negative feedback loop and de phosphorylation of $W_{lp}V_l$
3. Photo adduct decay of W_lV_l and $W_{lp}V_l$ heterodimer complex
4. Degradation W_lV_l and $W_{lp}V_l$

$$\frac{d[W_lV_l]}{dt} = k_9[W_l][V_l] - k_8[W_lV_l] - k_{\mathrm{pos4}}\frac{[W_lV_l][FFC]}{(K_{\mathrm{pos4}} + [W_lV_l])} + k_{\mathrm{dpos4}}[W_{lp}V_l] - k_{\mathrm{d5}}[W_lV_l] - k_5[W_lV_l] \tag{9}$$

$$\frac{d[W_{lp}V_l]}{dt} = k_{\mathrm{pos4}}\frac{[W_lV_l][FFC]}{(K_{\mathrm{pos4}} + [W_lV_l])} - k_{\mathrm{dpos4}}[W_{lp}V_l] - k_{\mathrm{dp4}}[W_{lp}V_l] - k_8[W_{lp}V_l] + k_9[W_{lp}][V_l] - k_5[W_{lp}V_l] \tag{10}$$

1.6 VVD dynamics

W_lW_l binds to the LRE region of the *vvd* gene and increases it's activity [16]. The VVD protein disrupts the WCC homodimerization by competitive binding to WC-1 in the presence of light, which acts as a feedback inhibitor of WCC [3, 7, 12, 17].

1. *vvd* transcription regulated by W_d, W_l, W_lW_l and W_lV_l. The paramater v_1 and K_2 are consider same for W_d, W_l, and W_lV_l mediated transciption.
2. Translation of *vvd* mRNA and VVD protein degradation
3. Conversion of V_d to V_l in the presence of light and degradation
4. Conversion of V_l to V_lV_l and degradation

$$\frac{d[mVVD]}{dt} = v_1\frac{[W_d]}{(K_2+[W_d])} + v_1\frac{[W_l]}{(K_2+[W_l])} + v_2\frac{[W_lW_l]}{(K_3+[W_lW_l])} + v_1\frac{[W_lV_l]}{(K_2+[W_lV_l])} - k_{\text{d6}}[mVVD] \tag{11}$$

$$\frac{d[V_d]}{dt} = v_3[mVVD] - l_1[light][V_d] + k_{10}[V_l] - k_{\text{d7}}[V_d] \tag{12}$$

$$\begin{aligned}\frac{d[V_l]}{dt} &= l_1[light][V_d] - k_{10}[V_l] + k_{11}[V_lV_l] - k_{12}[V_l]^2 - k_{\text{d8}}[V_l] \\ &+ k_8[W_lV_l] - k_9[W_l][V_l] + k_5[W_lV_l] + k_5[W_{lp}V_l] + k_8[W_{lp}V_l] - k_9[W_{lp}][V_l]\end{aligned} \tag{13}$$

$$\frac{d[V_lV_l]}{dt} = k_{12}[V_l]^2 - k_{11}[V_lV_l] - k_{\text{d9}}[V_lV_l] \tag{14}$$

1.7 FRQ dynamics

The W_d controls the transcription of the *frq* gene by binding to its clock box (C-box) in the dark which is essential and sufficient for rhythmic expression of *frq* [18]. The W_lW_l binds to the light responsive element (LRE) of *frq* in the presence of light and promote the *frq* expression [19, 20, 21]. The FRQ protein phosphorylate and transport to the nucleus [22, 23, 24, 25, 26]. WCC is hypophosphorylated and transcriptionally active in the absence of FRQ and shows opposite effect when FRQ is presence [27]. The FRQ protein inhibits WCC activity, forming a negative feedback loop [28].

1. *frq* transcription regulated by W_d, W_l, W_lW_l and W_lV_l. The paramater v_4 and K_4 are consider same for W_d, W_l, and W_lV_l mediated transciption.
2. Translation of inactive form FRQ and its degradation
3. In general the conversion of inactive FRQ to FCC is a complex process which involve series of reactions. Here, we introduce five steps from inactive FRQ to mature FCC with same rate constant k_{13}.

   $frqmRNA \xrightarrow{\text{v6}} FRQ \xrightarrow{\text{k13}} FRQ_1 \xrightarrow{\text{k13}} FRQ_2 \xrightarrow{\text{k13}} FRQ_3 \xrightarrow{\text{k13}} FRQ_4 \xrightarrow{\text{k13}} FRQ_5 \xrightarrow{\text{k13}} FFC \xrightarrow{\text{kd12}} \theta$ where the final FFC promotes the phosphorylation of WCC.

$$\frac{d[mFRQ]}{dt} = v_4\frac{[W_d]}{(K_4+[W_d])} + v_4\frac{[W_l]}{(K_4+[W_l])} + v_5\frac{[W_lW_l]}{(K_5+[W_lW_l])} + v_4\frac{[W_lV_l]}{(K_4+[W_lV_l])} - k_{\text{d10}}[mFRQ] \tag{15}$$

$$\frac{d[F_I]}{dt} = v_6[mFRQ] - k_{\text{d11}}[FRQ] \tag{16}$$

$$\frac{d[FRQ_1]}{dt} = k_{13}([FRQ] - [FRQ_1]) \tag{17}$$

$$\frac{d[FRQ_i]}{dt} = k_{13}([FRQ_{i-1}] - [FRQ_i]), i = 2, ..., 5 \tag{18}$$

$$\frac{d[FFC]}{dt} = k_{13}[FRQ_5] - k_{\mathrm{d12}}[FFC] \tag{19}$$

1.8 CSP-1 dynamics

CSP-1 is a global circadian repressor that is regulated by WCC, and it is essential for glucose compensation [29]. Additionally, CSP-1 inhibits its own transcription and *wc-1* in a glucose dependent manner [5, 29]. Here, given the very short lifetime of *csp-1* mRNA, we modelled the regulation of CSP-1 expression by W_d, W_l, W_lW_l, W_lV_l and inhibition of its own protein. Further, the parameter v_7, K_6 are considered the same for W_d, W_l, and W_lV_l mediated transcription and its own inhibition.

$$\begin{aligned}\frac{d[CSP]}{dt} &= v_7 \frac{[W_d]}{(K_6 + [W_d])} \frac{K_{\mathrm{cp2}}}{(K_{\mathrm{cp_2}} + [CSP])} + v_7 \frac{[W_l]}{(K_6 + [W_l])} \frac{K_{\mathrm{cp2}}}{(K_{\mathrm{cp_2}} + [CSP])} \\ &+ v_8 \frac{[W_lW_l]}{(K_7 + [W_lW_l])} \frac{K_{\mathrm{cp3}}}{(K_{\mathrm{cp_3}} + [CSP])} \\ &+ v_7 \frac{([W_lV_l]}{(K_6 + [W_lV_l])} \frac{K_{\mathrm{cp2}}}{(K_{\mathrm{cp_2}} + [CSP])} - k_{\mathrm{d13}}[CSP]\end{aligned} \tag{20}$$

1.9 FAM-3 dynamics

CSP-1 effectively represses the transcription of *fam-3* (ncu09497) gene, so that *fam-3* expression shows anti-phasic behaviour compared to *frq* or *csp-1* [29]. Here, we assumed the basal level of transcriptional activation of *fam-3* is negatively regulated by CSP-1.

$$\frac{d[mFAM]}{dt} = K_{\mathrm{basal1}} + v_9 \frac{K_{\mathrm{cp4}}}{(K_{\mathrm{cp_4}} + [CSP])} - k_{\mathrm{d14}}[mFAM] \tag{21}$$

1.10 Inference of transcription dynamics from luciferase time series

To estimate the unknown parameter of the *Neurospora crassa* circadian clock in LD condition, we inferred the mRNA dynamics from unstable luciferase (lucPEST) signals of *frq, vvd, csp-1* and *fam-3* measured under constant low light. We fitted the dense time-resolved luciferase activity to infer the mRNA dynamics based on the following equation:

$$\frac{d[LFRQ]}{dt} = vLf(v_4 \frac{[W_d]}{(K_4 + [W_d])} + v_4 \frac{[W_l]}{(K_4 + [W_l])} + v_5 \frac{[W_lW_l]}{(K_5 + [W_lW_l])} + v_4 \frac{[W_lV_l]}{(K_4 + [W_lV_l])}) - k_{\mathrm{dl}}[LFRQ] \tag{22}$$

$$\frac{d[LVVD]}{dt} = vLv(v_1 \frac{[W_d]}{(K_2 + [W_d])} + v_1 \frac{[W_l]}{(K_2 + [W_l])} + v_2 \frac{[W_lW_l]}{(K_3 + [W_lW_l])} + v_1 \frac{[W_lV_l]}{(K_2 + [W_lV_l])}) - k_{\mathrm{dl}}[LVVD] \tag{23}$$

$$\frac{d[LCSP]}{dt} = vLc(v_7 \frac{[W_d]}{(K_6 + [W_d])} \frac{K_{\text{cp2}}}{(K_{\text{cp}_2} + [CSP])} + v_7 \frac{[W_l]}{(K_6 + [W_l])} \frac{K_{\text{cp2}}}{(K_{\text{cp}_2} + [CSP])}$$
$$+ v_8 \frac{[W_l W_l]}{(K_7 + [W_l W_l])} \frac{K_{\text{cp3}}}{(K_{\text{cp}_3} + [CSP])} \quad (24)$$
$$+ v_7 \frac{([W_l V_l]}{(K_6 + [W_l V_l])} \frac{K_{\text{cp2}}}{(K_{\text{cp}_2} + [CSP])}) - k_{\text{dl}}[LCSP]$$

$$\frac{d[LFAM]}{dt} = vLfm(K_{\text{basal1}} + v_9 \frac{K_{\text{cp4}}}{(K_{\text{cp}_4} + [CSP])}) - k_{\text{dl}}[LFAM] \quad (25)$$

2 PARAMETER INFERENCE

The aim of parameter inference is to find the optimal sets of parameters for a given set of measurement data points, assuming the model M can describe the data sufficiently. To derive the unknown model parameters for the circadian clock, we used the standard method of minimizing the χ^2 value (weighted sum of squared residuals),

$$\chi^2(\theta) = \sum_{i=1}^{N} \left(\frac{y_i - y(t_i, \theta)}{\sigma_i} \right)^2 \quad (26)$$

where θ is the parameter vector; y_i and σ_i are measured mean value and standard deviation at time t_i, respectively; $y(t_i, \theta)$ is the simulated result from the model. The parameter inference was performed using the package Data2Dynamics (d2d)[30]. We used the Latin Hyper Cube sampling (700 samples) to scan the initial parameter space. Luciferase reporter data of several clock genes in WT and Δvvd were jointly used to infer the parameters (S3 Table, S4 Table).

REFERENCES

1 .Görl M, Merrow M, Huttner B, Johnson J, Roenneberg T, Brunner M. A pest-like element in frequency determines the length of the circadian period in neurospora crassa. *The EMBO journal* **20** (2001) 7074–7084.

2 .Lauinger L, Diernfellner A, Falk S, Brunner M. The rna helicase frh is an atp-dependent regulator of ck1a in the circadian clock of neurospora crassa. *Nature communications* **5** (2014) 1–10.

3 .Malzahn E, Ciprianidis S, Káldi K, Schafmeier T, Brunner M. Photoadaptation in neurospora by competitive interaction of activating and inhibitory lov domains. *Cell* **142** (2010) 762–772.

4 .Cesbron F, Brunner M, Diernfellner AC. Light-dependent and circadian transcription dynamics in vivo recorded with a destabilized luciferase reporter in neurospora. *PLoS One* **8** (2013) e83660.

5 .Sancar G, Sancar C, Brunner M. Metabolic compensation of the neurospora clock by a glucose-dependent feedback of the circadian repressor csp1 on the core oscillator. *Genes & development* **26** (2012) 2435–2442.

6 .Li C, Cesbron F, Oehler M, Brunner M, Höfer T. Frequency modulation of transcriptional bursting enables sensitive and rapid gene regulation. *Cell systems* **6** (2018) 409–423.

7 .Gin E, Diernfellner AC, Brunner M, Höfer T. The neurospora photoreceptor vivid exerts negative and positive control on light sensing to achieve adaptation. *Molecular systems biology* **9** (2013) 667.

8 .Talora C, Franchi L, Linden H, Ballario P, Macino G. Role of a white collar-1–white collar-2 complex in blue-light signal transduction. *The EMBO journal* **18** (1999) 4961–4968.

9 .Ballario P, Talora C, Galli D, Linden H, Macino G. Roles in dimerization and blue light photoresponse of the pas and lov domains of neurospora crassa white collar proteins. *Molecular microbiology* **29** (1998) 719–729.

10 .Froehlich AC, Liu Y, Loros JJ, Dunlap JC. White collar-1, a circadian blue light photoreceptor, binding to the frequency promoter. *Science* **297** (2002) 815–819.

11 .Cheng P, Yang Y, Wang L, He Q, Liu Y. White collar-1, a multifunctional neurosporaprotein involved in the circadian feedback loops, light sensing, and transcription repression of wc-2. *Journal of Biological Chemistry* **278** (2003) 3801–3808.

12 .Chen CH, DeMay BS, Gladfelter AS, Dunlap JC, Loros JJ. Physical interaction between vivid and white collar complex regulates photoadaptation in neurospora. *Proceedings of the National Academy of Sciences* **107** (2010) 16715–16720.

13 .Wu C, Yang F, Smith KM, Peterson M, Dekhang R, Zhang Y, et al. Genome-wide characterization of light-regulated genes in neurospora crassa. *G3: Genes, Genomes, Genetics* (2014) g3–114.

14 .He Q, Cheng P, Yang Y, Wang L, Gardner KH, Liu Y. White collar-1, a dna binding transcription factor and a light sensor. *Science* **297** (2002) 840–843.

15 .Wang B, Kettenbach AN, Gerber SA, Loros JJ, Dunlap JC. Neurospora wc-1 recruits swi/snf to remodel frequency and initiate a circadian cycle. *PLoS genetics* **10** (2014) e1004599.

16 .Heintzen C, Loros JJ, Dunlap JC. The pas protein vivid defines a clock-associated feedback loop that represses light input, modulates gating, and regulates clock resetting. *Cell* **104** (2001) 453–464.

17 .Hunt SM, Thompson S, Elvin M, Heintzen C. Vivid interacts with the white collar complex and frequency-interacting rna helicase to alter light and clock responses in neurospora. *Proceedings of the National Academy of Sciences* **107** (2010) 16709–16714.

18 .Froehlich AC, Loros JJ, Dunlap JC. Rhythmic binding of a white collar-containing complex to the frequency promoter is inhibited by frequency. *Proceedings of the National Academy of Sciences* **100** (2003) 5914–5919.

19 .Cheng P, Yang Y, Liu Y. Interlocked feedback loops contribute to the robustness of the neurospora circadian clock. *Proceedings of the National Academy of Sciences* **98** (2001) 7408–7413.

20 .He Q, Liu Y. Molecular mechanism of light responses in neurospora: from light-induced transcription to photoadaptation. *Genes & development* **19** (2005) 2888–2899.

21 .Dunlap JC. Proteins in the neurospora circadian clockworks. *Journal of Biological Chemistry* **281** (2006) 28489–28493.

22 .Garceau NY, Liu Y, Loros JJ, Dunlap JC. Alternative initiation of translation and time-specific phosphorylation yield multiple forms of the essential clock protein frequency. *Cell* **89** (1997) 469–476.

23 .Luo C, Loros JJ, Dunlap JC. Nuclear localization is required for function of the essential clock protein frq. *The EMBO Journal* **17** (1998) 1228–1235.

24 .Liu Y, Loros J, Dunlap JC. Phosphorylation of the neurospora clock protein frequency determines its degradation rate and strongly influences the period length of the circadian clock. *Proceedings of the National Academy of Sciences* **97** (2000) 234–239.

25 .Diernfellner AC, Querfurth C, Salazar C, Höfer T, Brunner M. Phosphorylation modulates rapid nucleocytoplasmic shuttling and cytoplasmic accumulation of neurospora clock protein frq on a circadian time scale. *Genes & development* **23** (2009) 2192–2200.

26 .Cha J, Yuan H, Liu Y. Regulation of the activity and cellular localization of the circadian clock protein frq. *Journal of Biological Chemistry* **286** (2011) 11469–11478.

27 .Schafmeier T, Haase A, Káldi K, Scholz J, Fuchs M, Brunner M. Transcriptional feedback of neurospora circadian clock gene by phosphorylation-dependent inactivation of its transcription factor. *Cell* **122** (2005) 235–246.

28 .Aronson BD, Johnson KA, Loros JJ, Dunlap JC. Negative feedback defining a circadian clock: autoregulation of the clock gene frequency. *Science* **263** (1994) 1578–1584.

29 .Sancar G, Sancar C, Brügger B, Ha N, Sachsenheimer T, Gin E, et al. A global circadian repressor controls antiphasic expression of metabolic genes in neurospora. *Molecular cell* **44** (2011) 687–697.

30 .Raue A, Steiert B, Schelker M, Kreutz C, Maiwald T, Hass H, et al. Data2dynamics: a modeling environment tailored to parameter estimation in dynamical systems. *Bioinformatics* **31** (2015) 3558–3560.
